# Magnetic Resonance Imaging and Flexible Hysterofiberscopic Findings of a Uterine Adenofibroma: Case Report and Literature Review

**DOI:** 10.1155/2018/9685683

**Published:** 2018-01-31

**Authors:** Hideki Watanabe, Naoya Harada, Ichiro Nobuhara, Noriko Haruta, Yumi Higashiura, Shioka Watanabe

**Affiliations:** Department of Obstetrics and Gynecology, Nara City Hospital, 1-50-1 Higashikidera-cho, Nara City, Nara Prefecture 630-8305, Japan

## Abstract

To our knowledge, highly detailed findings of flexible hysterofiberscopy in patients with adenofibroma have not been described. A 75-year-old nulliparous asymptomatic woman presented with a uterine polyp, which exhibited punctate heterogeneous hyperintensity or islands of isointense-to-hypointense signals on T2-weighted magnetic resonance imaging (MRI), hypointense signals on T1-weighted images (T1WI), and a little enhancement on contrast-enhanced T1WI. Flexible hysterofiberscopy revealed a red-pink polyp with a white-yellow, cobblestone-like surface easily deformed by perfusion fluid. The tumor was diagnosed histologically as an adenofibroma. Total abdominal hysterectomy and bilateral salpingo-oophorectomy were performed. The tumor in the uterus was necrotic macroscopically and histologically, and a residual adenofibroma could not be confirmed. At present, two years after surgery, the patient remains healthy. MRI and hysterofiberscopy can reveal the histological features of uterine adenofibromas and be useful for their diagnosis.

## 1. Introduction

Adenofibroma is an extremely rare benign biphasic neoplasm classified as an epithelial and mesenchymal tumor. Only approximately 30 cases have been reported to date (Table 1) [1–18]. Attempts have been made to diagnose these tumors using ultrasonography, computed tomography (CT), magnetic resonance imaging (MRI), hysteroscopy, and/or biopsy, but these methods were successful in correctly identifying adenofibroma in only three patients [3, 16, 17]. To date, only three patients each were assessed using MRI [11, 12, 14] and hysteroscopy [5, 15, 16]. We encountered a patient diagnosed histologically with adenofibroma using MRI and flexible hysterofiberscopy. To our knowledge, this is the first patient with adenofibroma who was assessed using both MRI and hysterofiberscopy. Highly detailed hysterofiberscopic findings in adenofibroma have not been reported previously. This report describes the clinical characteristics of this patient, including findings on MRI and flexible hysterofiberscopy, and provides a literature review of the clinical features of this rare neoplasm.

## 2. Case Report

**Figure 1:**
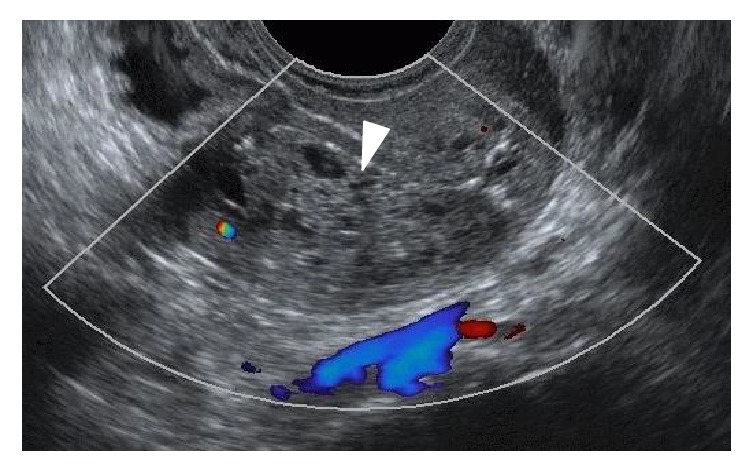
Figures on transvaginal color Doppler ultrasonography, showing a mass in the uterine cavity, measuring 3 × 2 cm and consisting of multiple cysts differing in size (triangle) and without pulsatile blood flow.

**Figure 2:**
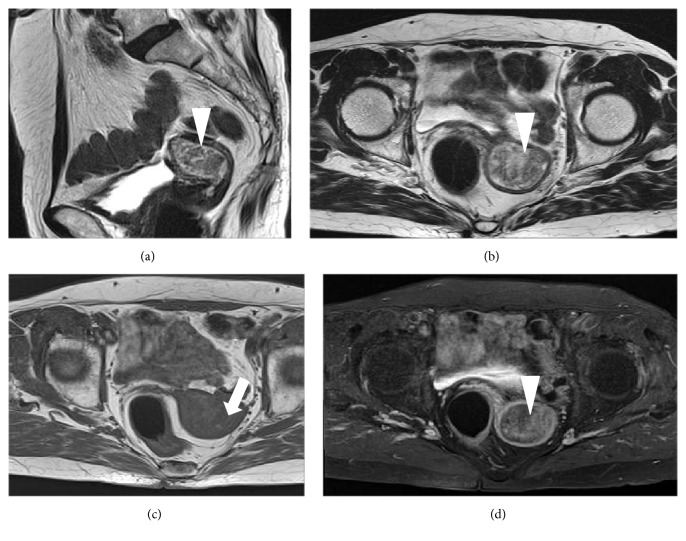
Magnetic resonance imaging of this patient. (a) Sagittal T2-weighted images (T2WI), showing an intrauterine tumor (triangle), which exhibited punctate heterogeneous hyperintensity or islands of isointense-to-hypointense signals. (b) View of the tumor (triangle) on axial T2WI. (c) Axial T1-weighted images (T1WI), showing that the tumor was detected as a hypointense signal with focal areas of high signal intensity (arrow), suspected of being hemorrhagic foci. (d) Axial contrast-enhanced T1WI showing a little tumor enhancement (triangle).

**Figure 3:**
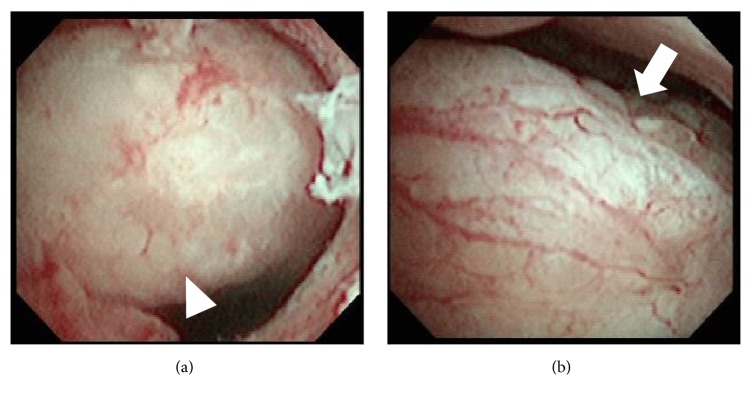
Hysterofiberscopy findings in this patient, showing (a) a red-yellow-white polyp with a partially reticulated surface (triangle) in the uterine cavity; (b) the lesion was easily deformed by perfusion fluid (arrow).

**Figure 4:**
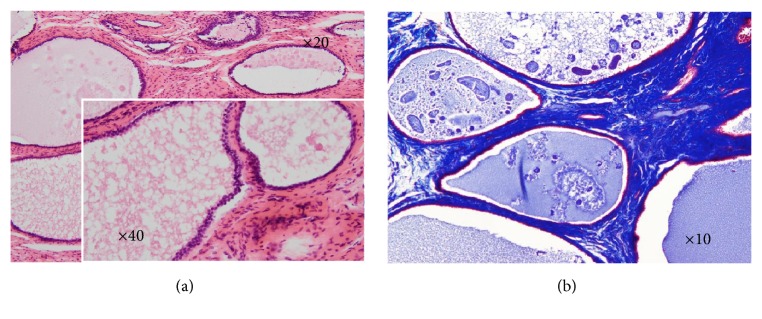
Microscopic findings, showing that (a) the tumor resulted from the benign biphasic proliferation of epithelial and mesenchymal components. The epithelial elements were endometrial glands of benign appearance (HE stain, objective magnification ×20). The inset shows that the mesenchymal component consisted of endometrial stroma containing fibroblasts of benign nuclear features and very low mitotic activity (HE stain, objective magnification ×40): (b) the mesenchymal part was strongly positive on Masson's trichrome staining, confirming the presence of collagen fibers (Masson's trichrome stain, objective magnification ×10).

**Figure 5:**
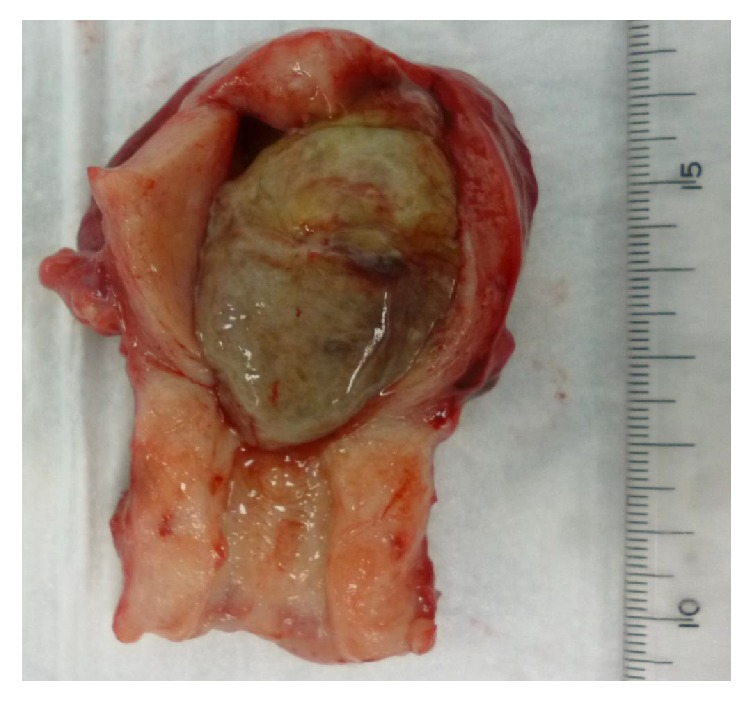
Macroscopic findings, showing a gray-semitransparent-edematous necrotic tumor, measuring approximately 4 × 2.5 cm, in the uterine cavity. The attached area could not be identified.

A 75-year-old nulliparous woman was referred to our hospital by a private internal medicine clinic for an asymptomatic intrauterine mass. She had been treated for type 2 diabetes mellitus and cholelithiasis for 20 years. The mass was detected on abdominal ultrasonography at the clinic. Transvaginal color Doppler ultrasonography at our hospital revealed a polypoid mass, measuring 3 × 2 cm, in the uterine cavity, and consisting of multiple low echogenic cysts that differed in size without pulsatile blood flow (Figure 1). The patient's serum CA125, CA19-9, CEA, SCC, and LDH concentrations were 11.2 U/mL, 6.7 U/mL, 2.7 ng/mL, 1.0 ng/mL, and 259 IU/mL, respectively. MRI also detected an intrauterine tumor, which exhibited punctate heterogeneous hyperintensity or islands of isointense-to-hypointense signals on T2-weighted images (T2WI) (Figures 2(a) and 2(b)). Axial T1-weighted images (T1WI) showed a hypointense signal with focal areas of high signal intensity, suspected of being hemorrhagic foci (Figure 2(c)), whereas axial contrast-enhanced T1WI showed insignificant enhancement (Figure 2(d)). The tumor was well-circumscribed without myometrial invasion, with no high-intensity areas on diffusion-weighted images. Flexible hysterofiberscopy revealed a reddish-pink polyp with a whitish-yellow, cobblestone-like surface (Figure 3(a)), easily deformed by perfusion fluid (Figure 3(b)). Transcervical exeresis was attempted using forceps, but only a small part of the tumor was removed because the cervix was insufficiently dilated. Microscopically, the tumor consisted of a benign biphasic proliferation of epithelial and mesenchymal components (Figure 4(a)). The epithelial elements were endometrial glands of benign appearance. The mesenchymal component was an endometrial stroma containing fibroblasts of benign nuclear features and very low mitotic activity. The mesenchymal part was strongly positive on Masson's trichrome staining, confirming the presence of collagen fibers (Figure 4(b)). Immunohistochemical staining showed that the stromal cells were negative for smooth muscle actin and CD10. These features suggested that the tumor was an adenofibroma. Hysterectomy was recommended, because an adenosarcoma may be present within the residual tumor or an adenofibroma may develop invasive potential and recur [4, 5]. Therefore, total abdominal hysterectomy and bilateral salpingo-oophorectomy were performed. Macroscopically, the mass in the uterus measured approximately 4 × 2.5 cm and was a gray-semitransparent-edematous necrotic tumor (Figure 5), although the attached area could not be identified. Histological examination revealed that the tumor was completely necrotic and amorphous, such that its origin could not be determined. Residual adenofibroma could not be confirmed in the uterus. One part of the endometrium contained an endometrial polyp, with a pathological morphology that differed completely from the adenofibroma. The postoperative course of the patient was uneventful, and no further treatment was required. Currently, two years after the surgery, the patient remains healthy.

## 3. Discussion

Endometrial adenofibroma was first described in 1959 as a benign form of mixed mesodermal tumor [1]. This rare type of noninvasive neoplasm is composed of benign epithelial and mesenchymal components, which can usually be sharply delineated from the underlying myometrium and adjacent endometrium. A similar type of tumor, Mullerian adenosarcoma, was first described in 1974 [19]. Mullerian adenosarcoma is a mixed mesodermal tumor in which the epithelium is benign, but the stromal component is histologically sarcomatous [19]. It is important to distinguish adenofibroma from adenosarcoma, as their expected clinical behavior differs. The most useful criterion for distinguishing adenofibroma from adenosarcoma is the frequency of mitotic figures in the stroma, with adenofibromas having three or fewer mitotic figures per 10 high-power fields (HPFs) and adenosarcomas having four or more mitotic figures per 10 HPFs [2]. Moreover, in contrast to adenosarcomas, adenofibromas do not have a marked degree of atypical mesenchymal cells, a histologically malignant heterologous element, or myometrial invasion [2]. However, one study described two adenofibromas that infiltrated deep into the myometrium, with one invading the lumen of myometrial veins [4]. The latter case resembles intravenous leiomyomatosis, a histologically benign leiomyoma derived from a uterine leiomyoma or intrauterine venous wall that grows and extends intravenously. The origin and malignant potential of these tumors remain unclear.

To date, approximately 30 cases of adenofibroma have been reported in the literature (considering only English publications) (Table 1) [1–18]. Adenofibromas occur primarily in postmenopausal women, of an average age of 57 years, but may also occur in women of reproductive age [17]. The chief complaints are usually abnormal vaginal bleeding and/or abdominal pain. About 90% of adenofibromas arise in the endometrium, with the other 10% reported to originate from the uterine endocervix. These tumors range in size from 2 to 14 cm, with an average diameter of 7.1 cm. Adenofibroma has been associated with tamoxifen therapy for breast cancer [10, 12, 18]. Tamoxifen is a selective estrogen receptor modulator widely used to treat patients with estrogen-dependent breast cancer. Tamoxifen is thought to act as a partial estrogen agonist on the endometrium, thereby increasing the incidence of proliferative endometrial lesions, including adenofibromas, polyp, endometrial hyperplasia, and endometrioid adenocarcinomas.

Although attempts have been made to diagnose adenofibroma using ultrasonography, CT, MRI, hysteroscopy, and/or biopsy, these methods were successful in only three patients [3, 16, 17]. To date, only three patients each have been evaluated using MRI [11, 12, 14] and hysteroscopy [5, 15, 16]. To our knowledge, this is the first patient who was diagnosed using both MRI and hysteroscopy. MRI findings in this patient were consistent with those previously described [11, 12, 14]. However, to our knowledge, highly detailed findings of flexible hysterofiberscopy have not been reported previously in patients with adenofibroma. Our findings of cobblestone-like surface and easy deformation by perfusion fluid reflect the histological features of adenofibroma with multilocular cysts containing secreted fluid.

Although endometrial adenofibromas are benign lesions, total hysterectomy is recommended, because these neoplasms may recur if incompletely curetted or locally excised [4, 5]. Hysterectomy assures complete excision, as well as permitting the thorough sampling needed to exclude the possibility of adenosarcoma. Indeed, most patients with adenofibroma underwent hysterectomy, with none showing tumor recurrence [1–14, 16–18].

Young women with adenofibroma may be given the option of lesion removal under hysteroscopic visualization, allowing retention of the uterus and reproductive potential. Surgical excision of adenofibromas in two patients, including one who underwent operative hysteroscopy with wide local excision, was successful, with no evidence of recurrence [15]. Recurrence associated with conservative treatment may be due to incomplete excision. Operative hysteroscopy with wide local excision may be considered an alternative to hysterectomy for women, who wish to preserve their reproductive function, provided that the completeness of excision is verified and long-term follow-up is possible.

The tumor in our patient was well demarcated, with no evidence of invasion into the myometrium on MRI. Necrotic tissue, likely the adenofibroma, was present in the uterine cavity after hysterectomy. Transcervical exeresis may have caused twisting of the tumor, resulting in necrosis. The tumor could be completely removed after hysterofiberscopy. The uterus did not contain any residual adenofibroma tissue, but the endometrium contained an endometrial polyp. Because the pathological morphology of these tumors differed completely, their causal relationship could not be determined.

## 4. Conclusion

Flexible hysterofiberscopy and MRI are also useful for the diagnosis of adenofibroma, which formed a polypoid mass in the uterine cavity.

## Figures and Tables

**Table 1:** List of previously described patients with uterine adenofibroma.

Authors [reference number]	Published year	Age (y)	Gravida	Para	Chief complaint	Locations	Maximum diameter (cm)	Initial examinations	Initial diagnosis	Treatment	Mitotic figures/10 HPF	Follow-up period (years)	Characteristics
Ober [1]	1959	NM	NM	NM	Abnormal vaginal bleeding	Endometrium	NM	NM	NM	TAH	NM	NM	

Zaloudek and Norris [2]	1981	46	NM	NM	Abnormal vaginal bleeding	Endometrium	NM	NM	NM	TAH, BSO	2	10	
		79	NM	NM	Abnormal vaginal bleeding	Endometrium	NM	NM	NM	TAH, BSO	2	9	
		71	NM	NM	Abnormal vaginal bleeding	Endometrium	NM	NM	NM	TAH, BSO	1	8	
		60	NM	NM	Abnormal vaginal bleeding	Endometrium	NM	NM	NM	TAH, BSO	3	5	
		62	NM	NM	Abnormal vaginal bleeding	Endometrium	NM	NM	Carcinosarcoma	TAH, BSO, radiation	2	4	
		73	NM	NM	Abnormal vaginal bleeding	Endometrium	NM	NM	NM	TAH, BSO	2	4	
		84	NM	NM	Abnormal vaginal bleeding	Endometrium	NM	NM	NM	TAH, BSO	1	1.5	
		48	NM	NM	Abdominal pain	Endometrium	NM	NM	NM	TAH, BSO	3	1	
		70	NM	NM	Abnormal vaginal bleeding	Endometrium	NM	NM	NM	TAH, BSO	1	Died of other disease	
		66	NM	NM	Abnormal vaginal bleeding	Endometrium	NM	NM	NM	TAH, BSO	2	Died of other disease	

Altaras et al. [3]	1984	78	1	1	Abnormal vaginal bleeding	Endometrium	8	Biopsy	Adenofibroma	TAH, BSO	0	NM	

Clement and Scully [4]	1990	70	2	2	Abnormal vaginal bleeding	Endometrium	4.5	Ultrasonography	Endometrial adenocarcinoma	TAH, BSO	<1	3.5	Myometrial invasion
		51	4	2	Abdominal pain, vomiting	Endometrium	NM	NM	NM	TAH, BSO	<1	3.25	Myometrial invasion, intravenous invasion

Seltzer et al. [5]	1990	28	0	0	Abnormal vaginal bleeding, abdominal pain	Endometrium	NM	Laparoscope, hysteroscope, biopsy	Endometrial polyp	TAH, LSO (after recurrence)	<1	3	Recurrence 2 years after excision

Agarwal et al. [6]	1991	38	NM	multi	Hypermenorrhea, low back pain	Endometrium, endocervix	12	NM	Uterine myoma	TAH	<1	2	Arising from the uterine body and the endocervix

Miller and McClure [7]	1992	68	2	2	Abnormal vaginal bleeding, abdominal pain	Endometrium	12	Ultrasonography, CT	Uterine myoma	TAH, BSO, radiation	0	0.75	Involvement of adenocarcinoma

Gemer et al. [8]	1995	48	NM	NM	None	Uterine serosa	6	Ultrasonography	NM	TAH, RSO	0	NM	Invasion through the uterine serosa to the right adnexa

Horie et al. [9]	1995	67	NM	0	Abdominal pain	Endometrium	6	Ultrasonography, CT	NM	TAH, BSO	NM	2	

Huang et al. [10]	1996	70	4	4	Abnormal vaginal bleeding	Endometrium	7	Ultrasonography, biopsy	Myomatous polyp	TAH, BSO	NM	NM	Treated with tamoxifen
		56	6	3	Abnormal vaginal bleeding	Endometrium	5.5	Ultrasonography, biopsy	NM	TAH	NM	NM	Treated with tamoxifen

Lee et al. [11]	1998	31	NM	0	Abdominal vaginal bleeding, hypermenorrhea	Endometrium	NM	CT, MRI, biopsy	Adenofibroma or adenosarcoma	TAH	NM	NM	

Oshima et al. [12]	2002	69	1	1	Abnormal vaginal bleeding	Endometrium	5	Ultrasonography, CT, MRI, biopsy	Endometrial polyp	TAH	NM	NM	Treated with tamoxifen

Haberal et al. [13]	2005	55	NM	NM	Abnormal vaginal bleeding, anemia	Endocervix	7	Ultrasonography, biopsy	NM	TAH, BSO	NM	NM	

Konishi et al. [14]	2006	42	NM	0	Abnormal vaginal bleeding, anemia	Endometrium	8	Ultrasonography, CT, MRI, biopsy	Mullerian mixed tumor	TAH, BSO	0	NM	

Bettaieb et al. [15]	2007	31	0	0	Abnormal vaginal bleeding	Endometrium	13	Ultrasonography	NM	Excision	<1	4	
		55	8	6	Abnormal vaginal bleeding	Endometrium	2	Ultrasonography, hysteroscope	NM	Excision with hysteroscope	<1	2	
		63	4	4	Uterine prolapse	Endometrium	5	NM	Uterine prolapse	TAH	<1	Not available	

Skorupskaite et al. [16]	2011	60	1	1	Abnormal vaginal bleeding, abdominal pain	Endometrium	4	Ultrasonography, hysteroscope, biopsy	Adenofibroma	TLH, RSO	<1	5	

Navada et al. [17]	2012	21	NM	NM	Abnormal vaginal bleeding, abdominal pain	Endocervix	14	Ultrasonography, CT, biopsy	Adenofibroma	TAH, BSO	0	NM	

Shi et al. [18]	2015	45	NM	NM	None	Endometrium, right ovary	6.5 & 6.5	Ultrasonography, biopsy	Endometrial hyperplasia	TAH, BSO	<1	1	Treated with tamoxifen

Present case		75	0	0	None	Endometrium	3	Ultrasonography, MRI, hysterofiberscopy, biopsy	Adenofibroma	TAH, BSO	<1	1	Coexisting endometrial polyp

NM, not mentioned; TAH, total abdominal hysterectomy; BSO, bilateral salpingo-oophorectomy; LSO, left salpingo-oophorectomy; RSO, right salpingo-oophorectomy; TLH, total laparoscopic hysterectomy; CT, computed tomography; MRI, magnetic resonance imaging; and HPF, high power field.
